# Spin defects in hBN as promising temperature, pressure and magnetic field quantum sensors

**DOI:** 10.1038/s41467-021-24725-1

**Published:** 2021-07-22

**Authors:** Andreas Gottscholl, Matthias Diez, Victor Soltamov, Christian Kasper, Dominik Krauße, Andreas Sperlich, Mehran Kianinia, Carlo Bradac, Igor Aharonovich, Vladimir Dyakonov

**Affiliations:** 1 Experimental Physics 6 and Würzburg-Dresden Cluster of Excellence ct.qmat, Julius Maximilian University of Würzburg, Würzburg, Germany; 2 Ioffe Institute, St. Petersburg, Russia; 3 School of Mathematical and Physical Sciences, University of Technology Sydney, Ultimo, NSW Australia; 4 Centre of Excellence for Transformative Meta-Optical Systems, University of Technology Sydney, Ultimo, NSW Australia; 5 Department of Physics & Astronomy, Trent University, Peterborough, ON Canada

**Keywords:** Electronic properties and materials, Qubits

## Abstract

Spin defects in solid-state materials are strong candidate systems for quantum information technology and sensing applications. Here we explore in details the recently discovered negatively charged boron vacancies (V_B_^−^) in hexagonal boron nitride (hBN) and demonstrate their use as atomic scale sensors for temperature, magnetic fields and externally applied pressure. These applications are possible due to the high-spin triplet ground state and bright spin-dependent photoluminescence of the V_B_^−^. Specifically, we find that the frequency shift in optically detected magnetic resonance measurements is not only sensitive to static magnetic fields, but also to temperature and pressure changes which we relate to crystal lattice parameters. We show that spin-rich hBN films are potentially applicable as intrinsic sensors in heterostructures made of functionalized 2D materials.

## Abstract (web summary)

Spin defects in two-dimensional materials potentially offer unique advantages for quantum sensing in terms of sensitivity and functionality. Here, the authors demonstrate the use of spin defects in hexagonal boron nitride as sensors of magnetic field, temperature and pressure, and show that their performance is comparable or exceeds that of existing platforms.

## Introduction

Spin defects in three-dimensional (3D) wide band-gap semiconductors have extensively been utilized in both fundamental and practical realizations in quantum science. The most prominent systems are the nitrogen-vacancy (NV) center in diamond^1^ and various types of spin defects in silicon carbide (SiC) (divacancy and silicon-vacancy)^2,3^. These systems reveal optically detected magnetic resonance (ODMR), which allows for polarization, manipulation, and optical readout of their spin state and consequent mapping of external stimuli (magnetic/electric field, temperature, pressure, etc.) onto it^4–6^. A variety of reports have demonstrated outstanding nanoscale sensing applications of NV-centers (particularly NV^−^) in physics and biology including detection of individual surface spins^7^ and nanothermometry in living cells^8,9^. However, NV-centers in diamond and spin centers in SiC possess intrinsic limitations. The three-dimensional nature of the material makes it challenging to position the spin-defects close as to the sample surface, and thus, to the object/quantity to be sensed. Furthermore, the proximity to the surface deteriorates their spin coherence properties and hinders their sensitivity as nano-sensors^10^.

A remedy to these limitations may be provided by recently discovered defects in layered materials. One of the most prominent stackable 2D materials is hexagonal boron nitride (hBN) which hosts a large variety of atom-like defects including single-photon emitters^11–14^. Spin carrying defects have been theoretically predicted and experimentally confirmed in hBN^15–19^. Currently, the most understood defect is the negatively-charged boron vacancy center (${{{{\rm{V}}}}}_{{{{\rm{B}}}}}^{-}$)^20^, which can be readily created by neutron irradiation, ion implantation, or femtosecond laser pulses^21,22^. Due to its spin-optical properties, the ${{{{\rm{V}}}}}_{{{{\rm{B}}}}}^{-}$ center is proving to be a promising candidate system for quantum information and nanoscale quantum sensing applications and has thus expanded the already large suite of unique features displayed by 2D materials^13^.

The recently identified ${{{{\rm{V}}}}}_{{{{\rm{B}}}}}^{-}$ in hBN displays a photoluminescence (PL) emission band around 850 nm and has been found to be an electronic spin-triplet (*S* = 1) system with a ground state zero-field splitting (ZFS) ${D}_{{gs}}/h\cong 3.5\,{{{\rm{GHz}}}}$ between its spin sublevels ${m}_{s}=0$ and ${m}_{s}=\pm 1$^16^. Here, we study the effect of external stimuli on the defect’s properties and demonstrate its suitability for sensing temperature, pressure (as lattice compression), and magnetic fields. Notably, our experiments show that the resolution and range of operation of the hBN ${{{{\rm{V}}}}}_{{{{\rm{B}}}}}^{-}$ center is competitive or exceeding those of similar defect-based sensors^23^.

## Results

**Fig. 1:**
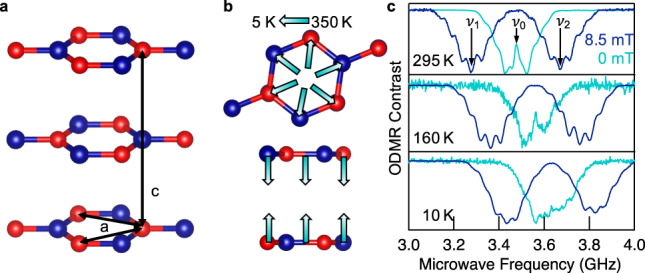
Schematic of the hexagonal boron nitride (hBN). **a** Alternating boron (red) and nitrogen (blue) atoms and the lattice constants *a* and *c*. **b** Lattice contraction and expansion due to temperature variation, according to crystallographic data^24^. **c** cw ODMR spectra measured with (dark blue) and without (cyan) external magnetic field at different temperatures *T* = 295, 160, and 10 K. Lowering of the temperature causes the resonances ${\nu }_{0},{\nu }_{1}$ and ${\nu }_{2}$ to shift to larger microwave frequencies indicating an increase of the zero-field splitting ${D}_{{gs}}$.

The results presented in this work were obtained on single-crystal hBN. The ${{{{\rm{V}}}}}_{{{{\rm{B}}}}}^{-}$ centers were generated in the sample via neutron irradiation ($\approx$2.3 × 10^18^ n cm^−2^), as described elsewhere^16,20^. More specifically, the absolute number of ${{{{\rm{V}}}}}_{{{{\rm{B}}}}}^{-}$ defects was determined as 10^13^ spins by electron paramagnetic resonance in the dark, giving the defect density of $\approx$5.4 × 10^17^ cm^−3^. Since we used neutron irradiation, it is assumed that the defects are homogeneously distributed in the sample. The hBN single-crystalline sample consists of a stack of a few thousand monolayers. The distance between two identically aligned layers is $c\cong 6.6\,\mathring{{{\rm{A}}}}$, while the in-plane distance between two identical atoms is ${{a}}\,{\mathtt{\cong }}\,2.5\,\mathring{{{\rm{A}}}}$ (Fig. 1a). As shown by temperature-dependent X-ray data^24^, the hBN lattice undergoes highly anisotropic thermal response with $c$ and *a* changing in opposite directions, i.e., while $c$ decreases with cooling, *a* increases, as schematically shown in Fig. 1b. This crystallographic feature can be used to monitor local temperature variations optically, via ODMR, since the temperature-driven compression/expansion of the lattice parameters *a* and $c$ causes a direct change in the ZFS parameter ${D}_{{gs}}$ of the triplet ground state^25^. Figure 1c shows continuous wave (cw) ODMR measurements for three different temperatures, with (dark blue) or without (cyan) an external magnetic field ${{{\boldsymbol{B}}}}$ applied. At room temperature and in the absence of the external magnetic field, the ODMR spectrum of the ${{{{\rm{V}}}}}_{{{{\rm{B}}}}}^{-}$ shows two resonances (${\nu }_{1},{\nu }_{2}$) centered symmetrically around ${\nu }_{0}$, which corresponds to the ZFS parameter ${D}_{{gs}}/h={\nu }_{0}=3.48\,{{{\rm{GHz}}}}$ with the splitting due to the non-zero off-axial ZFS parameter ${E}_{{gs}}/h\cong 50\,{{{\rm{MHz}}}}$. When applying an external static magnetic field ${{{\boldsymbol{B}}}}$, ${\nu }_{1}$ and ${\nu }_{2}$ split further following:

1 $${\nu }_{1,2}={D}_{{gs}}/h\pm \left(1/h\right)\sqrt{{E}_{{gs}}^{2}+{\left(g{\mu }_{{{\mathrm{B}}}}B\right)}^{2}}.$$

Here, *h* is Planck’s constant, *g* is the Landé factor and ${\mu }_{{{\mathrm{B}}}}$ is the Bohr magneton. The separation of the two resonances ${\nu }_{1}$ and ${\nu }_{2}$ can clearly be seen in Fig. 1c (dark blue traces). The visible substructure in both ODMR peaks is due to the hyperfine coupling of the electron *S* = 1 spin system (negatively charged boron vacancy) with three equivalent nearest nitrogen atoms, each possessing nuclear spin $I=1$ for the most abundant ^14^N isotope (99.63%). In total, seven hyperfine peaks can be resolved, whose relative separations are temperature and magnetic field independent. A closer look at Fig. 1c reveals that cooling down the sample results in a shift of the ODMR peaks to higher frequencies. Thus, the dependencies of the ODMR spectrum on temperature and magnetic fields can provide a basis for the use of the ${{{{\rm{V}}}}}_{{{{\rm{B}}}}}^{-}$ center as a thermometer and magnetometer at the sub-nanoscale.

### Temperature sensing

The observed shift of the resonances to higher frequency values (Fig. 1c) is independent of the applied magnetic field and is solely due to a reduction of the ZFS parameter ${D}_{{gs}}$. Over the temperature range 295−10 K, ${D}_{{gs}}$ undergoes a variation $\triangle {D}_{{gs}}\cong 195\,{{{\rm{MHz}}}}$. This is a relatively large change compared to analogous spin systems in 3D materials ($\approx$30-fold). For instance, the NV^−^ center in diamond exhibits a shift $\triangle {D}_{{gs}}\cong 7\,{{{\rm{MHz}}}}$^25^, while the ${D}_{{gs}}$ of V_Si_ in SiC is almost constant over the same range. Only more complex spin defects such as Frenkel defects (V_Si_−Si_i_) in SiC display a comparably strong effect ($\triangle {D}_{{gs}}\cong 300\,{{{\rm{MHz}}}}$)^5^.

**Fig. 2:**
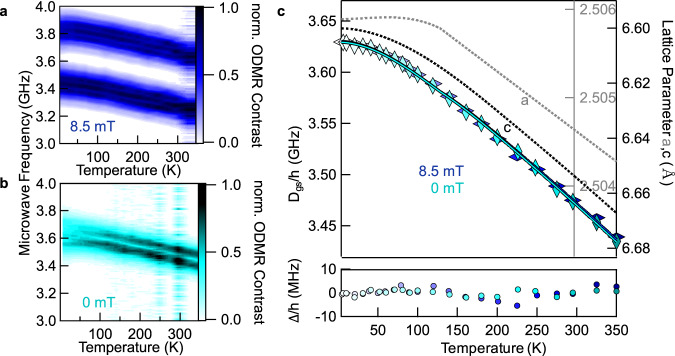
Temperature dependence of the ODMR spectrum of V^−^_B_. Color maps represent the peak positions of the normalized ODMR spectrum for different temperatures in an external magnetic field of **a** *B* = 8.5 mT and **b** *B* = 0 mT. **c** ZFS parameter ${{D}}_{{gs}}/{{h}}$ obtained from **a** (blue diamonds) and **b** (cyan diamonds) vs. temperature. The monotonic increase while lowering the sample temperature is unaffected by the magnetic field. The data can be fitted using Eq. (2) describing the temperature-dependent change of the lattice parameters *a* (gray dotted line^24^) and *c* (black dotted line^24^) that are also plotted in (**c**). The fits are shown as solid lines (blue for 8.5 mT and cyan for 0 mT) on top of the diamonds and reproduce the temperature dependence perfectly. (bottom panel) The difference between measurements and fit (Δ*/h*).

To quantify this temperature-induced shift of the ground state triplet energy-levels we combine temperature- and magnetic field-dependent ODMR measurements. Figure 2 summarizes the shift of ${D}_{{gs}}$ in the ODMR spectrum as a function of temperature both, in the presence (a) and absence (b) of an external magnetic field. In Fig. 2a, an external magnetic field of 8.5 mT is applied. A monotonic, nearly linear increase of the resonance frequencies associated to a change in the ZFS parameter ${D}_{{gs}}$ can be observed for temperatures down to 50 K. Zero-field ODMR (Fig. 2b) shows the same behavior. From Fig. 2a, b we now extract the ZFS values ${D}_{{gs}}/h$ and plot them against temperature (Fig. 2c). Both temperature dependencies, represented by dark blue and cyan diamonds, match perfectly and thus confirm that the temperature scaling of ${D}_{{gs}}$ is indeed independent of the magnetic field.

The temperature dependency can be explained by considering the change in the delocalization of the spin-defect wave function due to temperature-induced structural deformations of the crystal lattice. This is consistent with e.g., the case of the NV^−^ center in diamond^25,26^. The latter shows a linear behavior for the shift of the ZFS associated to the relative change of the lattice constant $\eta$ is observed^26^: $\triangle {D}_{{gs}}(\eta )/h=\theta \eta (T)$, where $\theta$ is the proportionality factor, explicitly written as ${{{{\mathrm{d}}}}D}/{{{\mathrm{d}}}}\eta$ and $\eta (T)$ is the relative change of the lattice parameter. Applying the same concept to hBN with its two lattice parameters ${{a}}$ and $c$ results in the equation:

2 $${{D}_{{gs}}\left(T\right)=D}_{{gs}}\left(295\,{{{\mathrm{K}}}}\right)+{\theta }_{{a}}{\eta }_{{a}}\left(T\right)h+{\theta }_{c}{\eta }_{c}(T)h$$

Here, ${D}_{{gs}}/h$ is the experimentally measured ZFS frequency. *D*_*gs*_(295 K)/*h* = 3.48 GHz is the ZFS at *T* = 295 K that we choose as reference, and ${\eta }_{{a}}(T)$, ${\eta }_{c}\left(T\right)$ are relative changes of ${{a}}$ and $c$, respectively (see also Eqs. (6) and (7) below). The temperature-dependent lattice parameters ${a}(T)$ and $c(T)$ for hBN were determined in ref. ^24^ and are plotted in Fig. 2c in addition to the ODMR data. The proportionality factors ${\theta }_{{a}}$ and ${\theta }_{c}$ are the significant parameters that connect lattice deformation and ZFS and will be derived from the experimental data in the following. To do so, Eq. (2) is fitted to the experimentally measured ZFS ${D}_{{gs}}$, as shown in Fig. 2c. The fit perfectly reproduces the experimental data, which highlights the remarkable linear response of the resonance frequency to changes of the lattice constants in this case due to temperature. More specifically, the maximum error between the fit and the measured data is <5 MHz, which is within the measured data scatter. Figure 2c (bottom panel) shows the difference between the fit and the measured data.

**Fig. 3:**
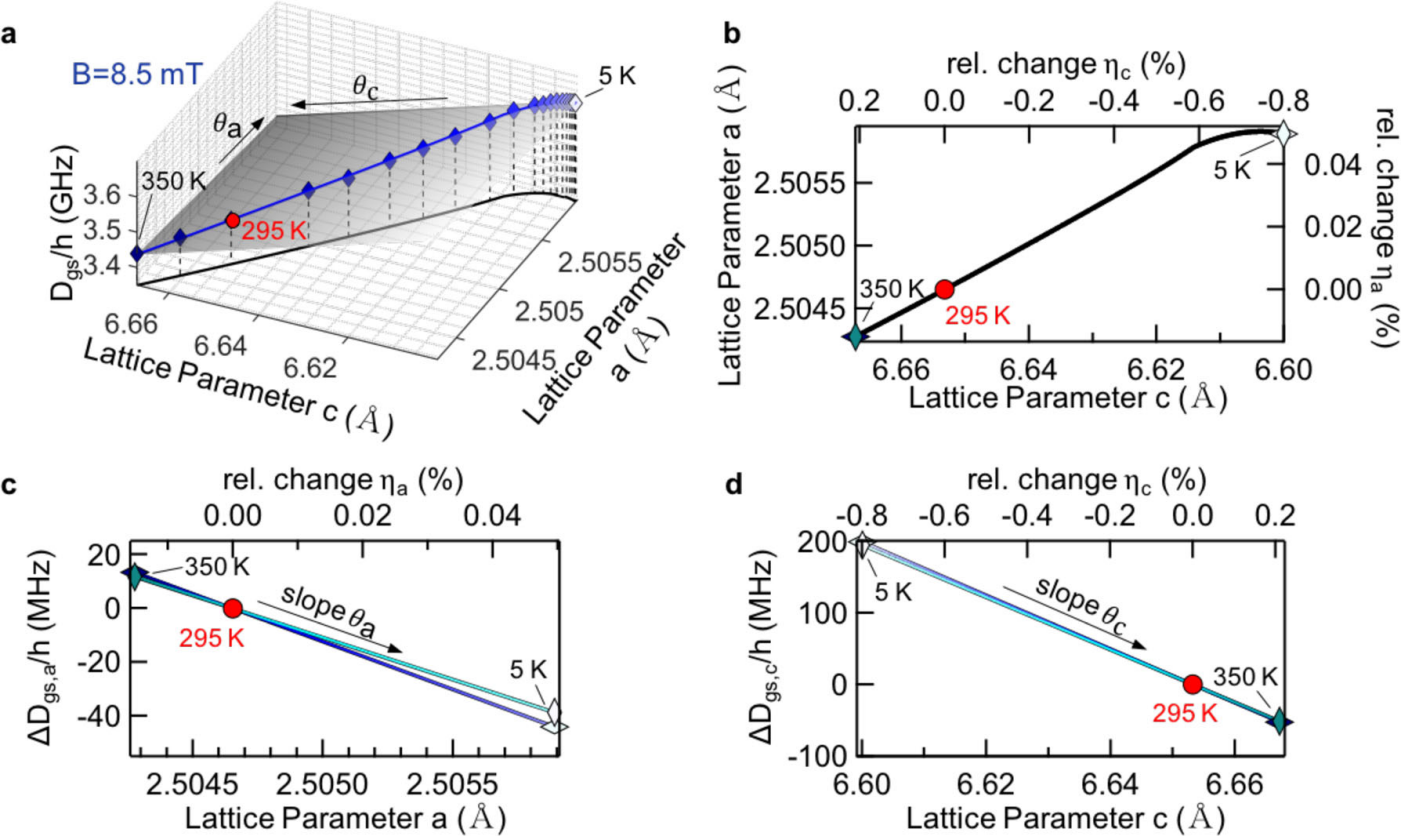
Zero-field splitting dependence on the lattice parameters *a* and *c*. **a** Combined three-dimensional representation of plots (**b**−**d**) with the slopes ${{\theta }}_{{a}}$and ${{\theta }}_{{c}}$ (shown also (**c**, **d**)). Equation (2) is fitted (solid lines) to the experimental data displayed as diamonds. The ZFS reference temperature *T* = 295 K is marked by the red dot. The assignment of colors of fitting lines (blue for 8.5 mT and cyan for 0 mT) is the same as in Fig. 2. **b** Comparison of lattice parameters in the temperature range 5−350 K. **c** Change of ZFS $\triangle {{{D}}}_{{{gs}},{{a}}}$ caused by the temperature-dependent lattice parameter ${{a}}$. **d** Change of ZFS $\triangle {{{D}}}_{{{gs}},{{c}}}$ caused by temperature-dependent lattice parameter *c*.

Figure 3 shows the relationship between temperature-dependent lattice parameters and ZFS by combining the three separate functions (${a}(T)$, $c(T)$ and ${D}_{{gs}}(T)$) of Fig. 2c in one plot (Fig. 3a). By inserting the crystallographic data for the hBN lattice parameters^24^ (Fig. 3b) into Eq. (2), we obtain partial shifts of the ZFS $\triangle {D}_{{gs},a}$ and $\triangle {D}_{{gs},c}$ with respective slopes ${\theta }_{{a}}$and ${\theta }_{c}$, as shown in Fig. 3c, d. For a more in-depth graphical analysis of the complex relationships, please refer to Supplementary Fig. 1.

We extract the values for ${\theta }_{{{a}}}$ of $(-84\pm 15)$ and $(-78.2\pm 8.8)\,{{{\rm{GH}}}}z$ or ${\theta }_{c}$ of $(-24.32\pm 0.59)$ and $(-24.6\pm 1.0)\,{{{\rm{GH}}}}z$ at *B* = 0 and 8.5 mT, respectively. The out-of-plane ${\theta }_{c}$ can be determined more precisely, since the relative change of lattice parameter $c$ is one order of magnitude larger. The values coincide within the experimental error and can be combined as:

3 $${\theta }_{{{a}}}=(-81+12)\,{{{\rm{GH}}}}z$$

4 $${\theta }_{c}=\left(-24.5{{{\rm{\pm }}}}0.8\right)\,{{{\rm{GH}}}}z$$

Remarkably, the ratio ${\theta }_{{{a}}}/{\theta }_{c}\approx$ 3.3, which means the influence of the lattice distortion on the ZFS in-plane is at least three times stronger than the influence of the interplanar distance on the same, indicating a localization of the ${{{{\rm{V}}}}}_{{{{\rm{B}}}}}^{-}$ spin density in the plane as predicted by the theory^17^.

Finally, we propose a polynomial, which allows a direct determination of ${D}_{{gs}}$ from the sample temperature $T$:

5 $${D}_{{gs}}\left(T\right)=h\mathop{\sum}\limits_{k}{A}_{k}{T}^{k}(k=0,1,2,3)$$

where *T* is the temperature, *h* is Planck’s constant, $k$ is an integer, and the polynomial coefficients ${A}_{k}$ are summarized in Table 1 for different temperature ranges. To obtain the coefficients ${A}_{k}$, the essential step is to determine the relative changes of the lattice parameters from the crystallographic data^24^ by using the Eqs. (6) and (7):

6 $${\eta }_{{{a}}}\left(T\right)=\frac{{{a}}\left(T\right){\mathtt{-}}{{a}}\left(295\,{{{\mathrm{K}}}}\right)}{{{a}}\left(295\,{{{\mathrm{K}}}}\right)}$$

7 $${\eta }_{c}\left(T\right)=\frac{c\left(T\right)-c\left(295\,{{{\mathrm{K}}}}\right)}{c\left(295\,{{{\mathrm{K}}}}\right)}$$

**Table 1:** Calculated polynomial coefficients ${{{A}}}_{{{k}}}$ for Eq. (5).

Temperature range (K)	${A}_{0}$ (GHz)	${A}_{1}$ (MHz K^−1^)	${A}_{2}$ (kHz K^−2^)	${A}_{3}$ (Hz K^−3^)
5–128	3.6367	0	−4.4308	11.468
128−189	3.6109	0.22839	−3.8805	5.522
189−350	3.6664	−0.55659	−0.2383	0

The three different temperature regions arise from the sectionally defined polynomial for the temperature-dependent lattice parameters *a* and ${{c}}$^24^. The exact procedure of calculation can be found in the Supplementary Information.

To estimate the heating effects induced by the laser excitation and resonant microwaves we performed ODMR measurements which are shown in Supplementary Figs. 2 and 3, respectively.

### Pressure sensing

The observation that a temperature-induced change in the lattice parameters directly results in a shift of the ZFS ${D}_{{gs}}$ leads to the consideration of utilizing the ${{{{\rm{V}}}}}_{{{{\rm{B}}}}}^{-}$ center also as a sensor, for externally applied in-plane or out-of-plane pressure. For a first-order estimation, we assume an isothermal system without shear strain and derive the perspective sensitivity based on reported elastic moduli for hBN crystals^27,28^. In cartesian coordinates, the pressure vector ${{{{\rm{\sigma }}}}}_{{xyz}}$ is given by the elastic moduli tensor $C$ multiplied with the relative change of the lattice parameters ${\eta }_{{xyz}}$:

8 $$\left(\begin{array}{c}{\sigma }_{x}\\ {\sigma }_{y}\\ {\sigma }_{z}\end{array}\right)=\left(\begin{array}{ccc}{C}_{11} & {C}_{12} & {C}_{13}\\ {C}_{12} & {C}_{11} & {C}_{13}\\ {C}_{13} & {C}_{13} & {C}_{33}\end{array}\right)\left(\begin{array}{c}{\eta }_{x}\\ {\eta }_{y}\\ {\eta }_{z}\end{array}\right)$$

The reported elastic moduli for hBN are: ${C}_{11}$ = (811 ± 12) GPa, ${C}_{12}$ = (169 ± 24) GPa, ${C}_{13}$ = (0 ± 3) GPa and ${C}_{33}$ = (27 ± 5) GPa^27^. The hBN lattice parameters *a* and $c$ are oriented along the *y*- and *z*-direction, respectively. This simplifies Eq. (8) by incorporating ${\eta }_{{{a}}{\mathtt{,}}c}$ and removing ${C}_{13}$, which is 0:

9 $$\left(\begin{array}{c}{\sigma }_{x}\\ {\sigma }_{y}\\ {\sigma }_{z}\end{array}\right)=\left(\begin{array}{c}{\eta }_{a}\left({\frac{2}{3}C}_{11}+{\frac{1}{3}C}_{12}\right)\\ {\eta }_{a}\left(\frac{2}{3}{C}_{12}+{\frac{1}{3}C}_{11}\right)\\ {\eta }_{c}{C}_{33}\end{array}\right)$$

This can be rewritten to obtain ${\eta }_{{{a}}{\mathtt{,}}c}$ directly:

10 $${\eta }_{a}={\sigma }_{x}/\left(\frac{2}{3}{C}_{11}+\frac{1}{3}{C}_{12}\right)={\sigma }_{y}/\left(\frac{2}{3}{C}_{12}+\frac{1}{3}{C}_{11}\right)$$

11 $${\eta }_{c}={\sigma }_{z}/{C}_{33}$$

Substituting these relationships into Eq. (2) yields the ZFS as a function of the applied pressure:

12 $${D}_{{gs}}({\sigma }_{x},{\sigma }_{y},{\sigma }_{z}) 	={D}_{{gs},295\,{{{\mathrm{K}}}}}+\triangle {D}_{{gs},x}+\triangle {D}_{{gs},y}+\triangle {D}_{{gs},z}\\ 	={D}_{{gs},295\,{{{\mathrm{K}}}}}+\frac{{\theta }_{a}{\sigma }_{x}h}{\tfrac{2}{3}\,{C}_{11}+\tfrac{1}{3}\,{C}_{12}}+\frac{{\theta }_{a}{\sigma }_{y}h}{\tfrac{2}{3}\,{C}_{12}+\tfrac{1}{3}\,{C}_{11}}+\frac{{\theta }_{c}{\sigma }_{z}h}{{C}_{33}}$$

Based on our estimates for ${\theta }_{{{a}}{\mathtt{,}}c}$ in Eqs. (3) and (4) and the reported elastic moduli^27^, we obtain the sensitivity to measure the ZFS shifts for each direction of the applied pressure:

13 $$\triangle {D}_{{gs},x}={\sigma }_{x}h(-0.136\pm 0.028)\,{{{\mathrm{Hz}}}}/{{{\mathrm{Pa}}}}$$

14 $$\triangle {D}_{{gs},y}={\sigma }_{y}h(-0.212\pm 0.052)\,{{{\mathrm{Hz}}}}/{{{\mathrm{Pa}}}}$$

15 $$\triangle {D}_{{gs},z}={\sigma }_{z}h(-0.91\pm 0.20)\,{{{\mathrm{Hz}}}}/{{{\mathrm{Pa}}}}$$

Consequently, we find that the ZFS shift $\triangle {D}_{{gs},{xyz}}$ is directly associated with external compression of the hBN lattice and therefore ${{{{\rm{V}}}}}_{{{{\rm{B}}}}}^{-}$ can be utilized as a pressure sensor. Remarkably, the out-of-plane sensitivity along the $c$-axis is much higher due to the small ${C}_{33}$ coefficient. This makes this type of sensor particularly useful to measure vertical indentation in 2D heterostructures.

**Fig. 4:**
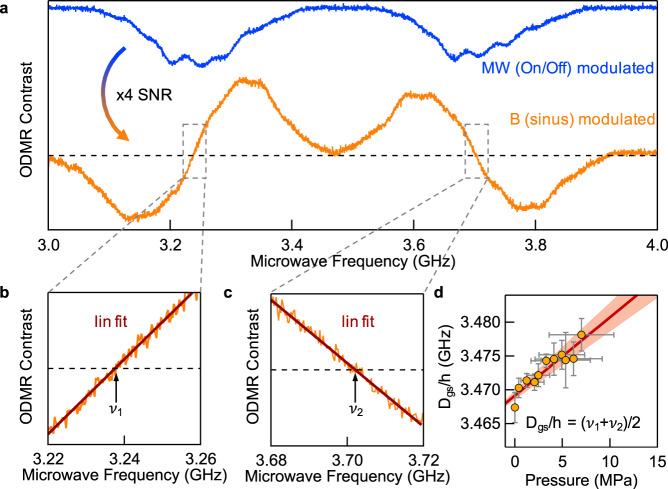
Pressure dependence of the zero-field splitting parameter *D*_*gs*_/*h*. **a** ODMR spectra of ${{{{\rm{V}}}}}_{{{{\rm{B}}}}}^{-}$ with microwave modulation (on/off) (blue) and sinusoidal magnetic field modulation (orange). The partially resolved hyperfine peaks are blurred due to an intentional overmodulation of the spectrum (for details see Supplementary Fig. 4) that allows a linear fit in the vicinity of the zero-crossings for a precise determination of the resonant transitions ${{{{\rm{\nu }}}}}_{1}$ (**b**) and ${{{{\rm{\nu }}}}}_{2}$ (**c**) and therefore the parameter ${{D}}_{{gs}}/{{h}}$. **d** ${{D}}_{{gs}}/{{h}}$ as a function of pressure, follows a slope of (1.16 ± 0.15) ${{{\rm{Hz}}}}/{{{\rm{Pa}}}}$ which is close to the expected value (0.91 ± 0.20) ${{{\rm{Hz}}}}/{{{\rm{Pa}}}}$ according to Eq. (15). Vertical error bars represent the standard deviations of the linear fits for ${{{{\rm{\nu }}}}}_{1}$and ${{{{\rm{\nu }}}}}_{2}$ in panels (**b**, **c**). Horizontal error bars indicate the uncertainty of the determination of the sample area to which the pressure is applied.

To verify the above considerations, we performed pressure-dependent experiments, which are shown in Fig. 4. Pressure is applied in the $c$-direction of the hBN lattice by stacking weights on the sample. To improve the sensitivity, we applied sinusoidal modulation to the static *B*-field instead of amplitude modulation (on/off) of the microwave while sweeping the frequency. The sine-wave modulation of the *B*-field yields the first derivative of the spectrum (see Supplementary Fig. 4 for the modulation behavior of the spectrum), which increases the signal-to-noise ratio by a factor of four (Fig. 4a, orange). For an accurate determination of the resonant transitions ${\nu }_{1}$ and ${\nu }_{2}$ and their shift with the applied pressure, the spectra were linearly fitted near the zero crossings, as shown in zoomed-in Fig. 4b, c. The resulting pressure dependence of the parameter ${D}_{{gs}}/h$ is shown in Fig. 4d. The experimentally determined slope of (1.16 ± 0.15) ${{{{\mathrm{Hz}}}}}/{{{{\mathrm{Pa}}}}}$ is close to the theoretically expected slope (0.91 ± 0.20) ${{{{\mathrm{Hz}}}}}/{{{{\mathrm{Pa}}}}}$ according to Eq. (15).

As we show in Supplementary Fig. 5, the method can be also potentially used to study local and temperature-induced strains in the sample by measuring the off-axis ZFS parameter *E*_*gs*_.

### Magnetic field sensing

**Fig. 5:**
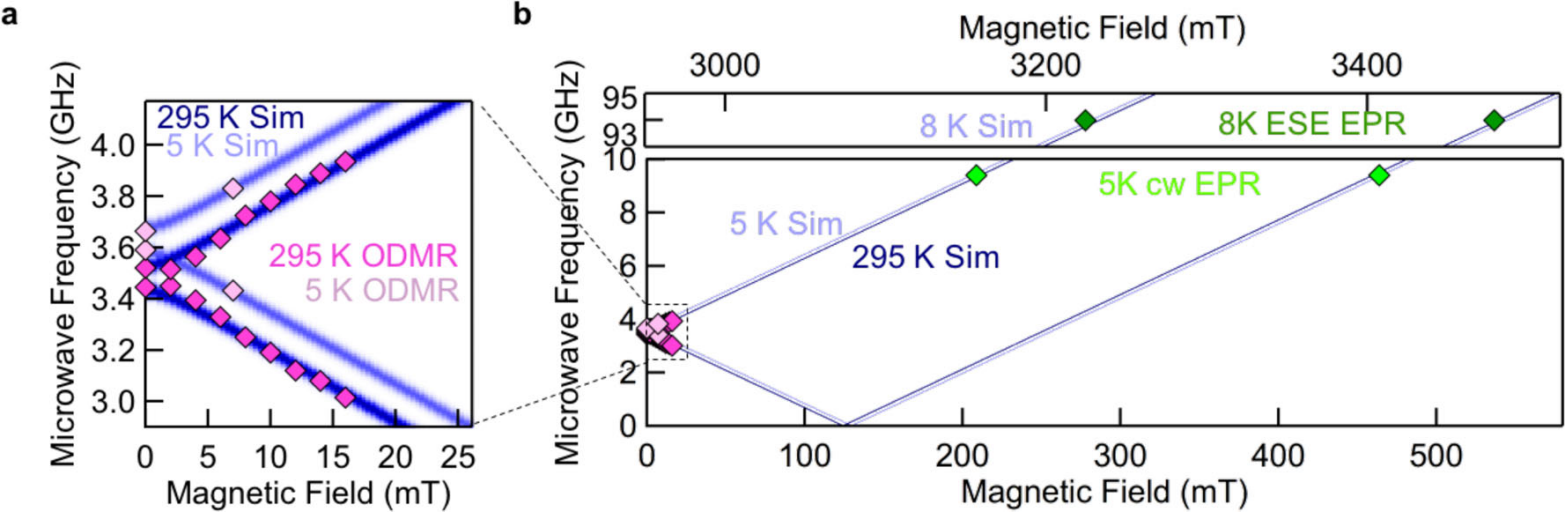
Experimental (diamonds) and simulated (dark and light blue traces) resonant frequencies of V^−^_B_ for different temperatures and magnetic fields. **a** ODMR measurements (pink diamonds) at *B* < 20 mT. **b** cw EPR measurement at *T* = 5 K and microwave frequency of 9.4 GHz (light green) and electron spin-echo measurements at *T* = 8 K and 94 GHz (dark green). Note the axes are shifted for better visibility and comparability.

As shown in Fig. 2a, the two resonant transitions ${\nu }_{1,2}$ are equally separated with respect to ${\nu }_{0}$ over the entire temperature range between 5 and 350 K. It should be pointed out that the magnetic field sensing is based on the g-factor, which is independent of the lattice parameters. In Fig. 5, we demonstrate the principle suitability of a ${{{{\rm{V}}}}}_{{{{\rm{B}}}}}^{-}$ center in hBN for magnetic field sensing, where we show the resonant microwave transitions ${\nu }_{1}$ and ${\nu }_{2}$ over a broad range (0−3500) mT and exemplarily simulated for two distant temperatures, *T* = 295 K (dark blue) and *T* = 5 K (light blue). For a magnetic field applied in the $c$-direction of the hBN lattice, the behavior can be described with Eq. (1). Due to the non-zero off-axis ${E}_{{gs}}$, the Zeeman splitting term $g{\mu }_{{{{\mathrm{B}}}}}B$ leads to a linear regime only for *B* > 3 mT, when the applied magnetic field is large enough to separate the two otherwise partially overlapping ${\nu }_{1,2}$ transitions.

To extend the magnetic field range of our measurements beyond the confocal ODMR setup limit of ≈20 mT, we applied cw electron paramagnetic resonance (cw EPR) and electron spin-echo detected (ESE) EPR. The advantage is that the cw EPR measurements are performed at a microwave frequency of $9.4\,{{{\rm{GHz}}}}$ (X-band) (light green diamonds) and the ESE EPR measurements at a microwave frequency of 94 GHz (W-band) (dark green diamonds) which allow extending the magnetic field range to 3500 mT. The multifrequency spin resonance approach enables us to determine the g-factor with extremely high accuracy as $g=2.0046\pm 0.0021$.

## Discussion

As we mentioned, one of the crucial parameters for high-sensitivity sensing is the distance between the sensor and the object to be sensed. In this regard, sensors based on the hBN ${{{{\rm{V}}}}}_{{{{\rm{B}}}}}^{-}$ center are particularly appealing as—based on the presented model of lattice constant variations—the required hardware ideally consists of only three hBN layers, with the intermediate one hosting a ${{{{\rm{V}}}}}_{{{{\rm{B}}}}}^{-}$ center. This corresponds to a minimum distance of the defect to the surface in the sub-nanometer range of ≈0.33 nm. A further thickness reduction, e.g., to a single monolayer, would indeed eliminate the interlayer contribution $c(T)$, but make the sensing effect completely dependent on the interaction of the ${{{{\rm{V}}}}}_{{{{\rm{B}}}}}^{-}$ wave function with the parameters of the adjacent material. For single layers, ${D}_{{gs},295\,{{{\mathrm{K}}}}}$ would differ from the theoretical calculation^29^, and a calibration would be required to determine the set of parameters (${D}_{{gs},295\,{{{{\mathrm{K}}}}}}$, ${\theta }_{a}$, ${\theta }_{c}$) specific for the adjacent material. To substantiate this hypothesis, however, further measurements and calculations must be carried out.

In the following, we benchmark the properties of the hereby proposed sensor based on ${{{{\rm{V}}}}}_{{{{\rm{B}}}}}^{-}$ centers in hBN against other defect-based sensors in silicon carbide and diamond. For this purpose, the general sensitivity is derived, which includes the respective coupling coefficient ${\Gamma }_{T,\sigma ,{B}}$ representing the sensitivity of the ODMR frequency shift due to the corresponding external influence, and the general resolution in relative change of frequency in relation to acquisition time and noise level^5^. An overview of all calculated coupling coefficients and resolutions is summarized in Table 2.

**Table 2:** Three spin hosting systems in comparison: coupling coefficient $\Gamma$ and general resolution ${{{\rm{\delta }}}}$ at room (*T* = 295 K) and cryogenic (*T* = 50 K) temperatures.

	Coupling coefficient $\Gamma$			Resolution $\delta$ (295 K)			Resolution $\delta$ (50 K)
	hBN	Diamond	SiC	hBN	Diamond	SiC	hBN
Magnetic field *B*	$g{\mu }_{{{{\mathrm{B}}}}}=28.0\,{{{\mathrm{kHz}}}}/\upmu{{{\mathrm{T}}}}$(for $g=2.00$)			$85.1\,\upmu {{{\mathrm{T}}}}/{\sqrt{{{\mathrm{Hz}}}}}$	$3\, {{{\mathrm{nT}}}}/{\sqrt{{{\mathrm{Hz}}}}}$	$10\,\upmu {{{\mathrm{T}}}}/{\sqrt{{{\mathrm{Hz}}}}}$	$4.33\,\upmu {{{\mathrm{T}}}}/{\sqrt{{{\mathrm{Hz}}}}}$
Temperature *T*	$-623\,{{{\mathrm{kHz}}}}/{{{\mathrm{K}}}}$	$-74\,{{{\mathrm{kHz}}}}/{{{\mathrm{K}}}}$	−1.1 MHz/K	$3.82\,{{{\mathrm{K}}}}/{\sqrt{{{\mathrm{Hz}}}}}$	$0.76\, {{{\mathrm{mK}}}}/{\sqrt{{{\mathrm{Hz}}}}}$	$1\, {{{\mathrm{K}}}}/{\sqrt{{{\mathrm{Hz}}}}}$	$0.19\, {{{\mathrm{K}}}}/{\sqrt{{{\mathrm{Hz}}}}}$
*X* Pressure ${\sigma }_{x}$	$-0.136\,{{{\mathrm{Hz}}}}/{{{\mathrm{Pa}}}}$			$17.5\cdot {10}^{6}\, {{{\mathrm{Pa}}}}/{\sqrt{{{\mathrm{Hz}}}}}$			$0.891\cdot {10}^{6}\, {{{\mathrm{Pa}}}}/{\sqrt{{{\mathrm{Hz}}}}}$
*Y* Pressure ${\sigma }_{y}$	$-0.212\,{{{\mathrm{Hz}}}}/{{{\mathrm{Pa}}}}$			$11.2\cdot {10}^{6}\, {{{\mathrm{Pa}}}}/{\sqrt{{{\mathrm{Hz}}}}}$			$0.572\cdot {10}^{6}\, {{{\mathrm{Pa}}}}/{\sqrt{{{\mathrm{Hz}}}}}$
*Z* Pressure ${\sigma }_{z}$	$-0.91\,{{{\mathrm{Hz}}}}/{{{\mathrm{Pa}}}}$			$2.62\cdot {10}^{6}\, {{{\mathrm{Pa}}}}/{\sqrt{{{\mathrm{Hz}}}}}$			$0.133\cdot {10}^{6}\, {{{\mathrm{Pa}}}}/{\sqrt{{{\mathrm{Hz}}}}}$

The table also shows the reference values for spin defects in diamond^30,32,33^ and SiC^5^, respectively.

In order to facilitate comparison with other color centers in 3D materials, we consider first a linear regime (50–350 K) in our analysis. In this range, a proportionality between $\triangle {D}_{{gs}}$ and $T$ is given by the factor ${\Gamma }_{T}=\tfrac{\triangle {D}_{{gs}}}{\triangle T}=-623\,{{{\mathrm{kHz}}}}/{{{\mathrm{K}}}}$. This value is almost one order of magnitude (> 8-fold) larger than the corresponding factor for NV^−^ centers in diamond ($-74\,{{{\mathrm{kHz}}}}/{{{\mathrm{K}}}}$)^30^. This remarkable difference is in particular due to the larger relative change of the lattice parameters as a function of temperature in hBN, while $\theta$ is of the same order of magnitude. For NV^−^ centers, a value of ${\theta }_{{{{{\mathrm{NV}}}}}}=-14.41\,{{{\rm{GHz}}}}$ is reported^26^, which is comparable to ${\theta }_{c}\approx -24\,{{{\rm{GHz}}}}$ for ${{{{\rm{V}}}}}_{{{{\rm{B}}}}}^{-}$ in hBN. Note that ${\theta }_{{a}}$ can be neglected here, since on the one hand the relative change of the lattice parameter *a* is negligible and on the other hand it counteracts the effect due to the expansion of the in-plane distance while cooling the sample. Interestingly, for temperatures below ~50 K, the quantity *D*_*gs*_ remains almost constant in NV^−^ centers in diamond^31^, which limits their operating range. Conversely, *D*_*gs*_*/h* in ${{{{\rm{V}}}}}_{{{{\rm{B}}}}}^{-}$ centers maintains a measurable temperature dependence well-below 50 K, down to a few K (see Fig. 2c). We estimate ${{{\mathrm{d}}}}{D}_{{gs}}/{{{{\mathrm{d}}}}T}(T=10\,{{{\mathrm{K}}}})=$ $-87\,{{{\mathrm{kHz}}}}/{{{\mathrm{K}}}}$ for hBN, while ${{{\mathrm{d}}}}{D}_{{gs}}/{{{{\mathrm{d}}}}T}(T=10\,{{{\mathrm{K}}}})$ in the NV^−^ diamond is in the range of $-7\times {10}^{-2}\,{{{\mathrm{kHz}}}}/{{{\mathrm{K}}}}$^31^, i.e., three orders of magnitude smaller. This is particularly intriguing for applications that require monitoring temperature changes, with high spatial resolution, in cryogenic conditions.

The general resolution ${\delta }_{T}^{295{{{\mathrm{K}}}}}$ obtained at room temperature is approximately $3.82\, {{{\mathrm{K}}}}/{\sqrt{{{\mathrm{Hz}}}}}$ which is of the same order of magnitude as defect-based temperature sensors in SiC ($1\, {{{\mathrm{K}}}}/{\sqrt{{{\mathrm{Hz}}}}}$) using the same cw ODMR set-up^5^. It should be noted, however, that at cryogenic temperatures the resolution of the ${{{{\rm{V}}}}}_{{{{\rm{B}}}}}^{-}$ center is enhanced by a factor of $\approx$20 (${\delta }_{T}^{50\,{{{\mathrm{K}}}}}=0.19\, {{{\mathrm{K}}}}/{\sqrt{{{\mathrm{Hz}}}}}$), as both the ODMR contrast ΔPL/PL and the PL intensity increase, which significantly reduces the required measurement time. Despite the smaller coupling coefficient, a temperature sensor based on NV’s in diamond is still more sensitive (0.76$\,\tfrac{{{{{\mathrm{mK}}}}}}{\sqrt{{{{{\mathrm{Hz}}}}}}}\,$)^32^, mainly due to stronger PL emission, higher ODMR contrast, and an optimized pulsed measurement protocol that exceeds the sensitivity of standard cw ODMR measurements performed here. Analogously, the magnetic field resolution of the ${{{{\rm{V}}}}}_{{{{\rm{B}}}}}^{-}$ can be quantified at room temperature as ${\delta }_{B}^{295{{{\mathrm{K}}}}}=85.1\,\upmu {{{\mathrm{T}}}}/{\sqrt{{{\mathrm{Hz}}}}}$(${\delta }_{B}^{50\,{{{\mathrm{K}}}}}=4.33\,\upmu {{{\mathrm{T}}}}/{\sqrt{{{\mathrm{Hz}}}}}$ at *T* = 50 K). This is comparable to V_Si_ in SiC ($10\,\upmu {{{\mathrm{T}}}}/{\sqrt{{{\mathrm{Hz}}}}}$)^5^ but lower than for NVs in diamond ($3\, {{{\mathrm{nT}}}}/{\sqrt{{{\mathrm{Hz}}}}}$)^33^. However, spin defects in 3D materials can lose their superior properties if they are close to the surface of the crystalline host^10^, which also leads to an inevitable limitation of the minimum achievable sensor-to-object distance. This can significantly hinder the effectiveness of the spin defects-based sensors in 3D materials, highlighting the potential advantages of the ${{{{\rm{V}}}}}_{{{{\rm{B}}}}}^{-}\,$ center as a nanometer-scale sensor.

Finally, we perform an estimate of the minimum detectable magnetic field, which is based on the shot noise limited sensitivity analysis^34^. Accordingly:

16 $$\delta B\cong \frac{1}{g{\mu }_{{{\mathrm{B}}}}R\sqrt{\eta }\sqrt{{Nt}{T}_{2}^{\ast }}},$$

where $g$ is the electronic Landé factor and ${\mu }_{{{\mathrm{B}}}}$ is the Bohr magneton, so that $g{\mu }_{{{\mathrm{B}}}}\approx 28\,{{{{\mathrm{MHz}}}}}/{{{{\mathrm{mT}}}}}$, the ODMR contrast for ${{{{\rm{V}}}}}_{{{{\rm{B}}}}}^{-}$ center is *R* = 0.1%, $\eta$ is the collection efficiency, $N$ is the number of active spins, and *t* = 1 s is taken as measurement time. Since the ${{{{\rm{V}}}}}_{{{{\rm{B}}}}}^{-}$ density is known and we excite a voxel of about 10 μm diameter, we can estimate a number $N\approx 2.6\times {10}^{9}$ of simultaneously addressed spins, and from the previously measured Rabi oscillations^20^, we derived the dephasing time *T*_2_* = 100 ns. The value for $\eta$ is limited by both the efficiency of the detection setup and the optical properties of the spin-hosting sample, and is therefore important. On one hand, the photon extraction efficiency of ${{{{\rm{V}}}}}_{{{{\rm{B}}}}}^{-}$ defects in hBN is very high (near-unity for hBN monolayers) compared to, e.g., that of spin systems such as color centers in diamonds. This is a consequence of the 2D nature of hBN which renders it less prone to phenomena such as total internal reflection or scattering. On the other hand, we work with crystals (even if the refractive index is lower than diamonds) and in our optical detection system, we use a lens with low numerical aperture NA = 0.3. This leads us to consider a conservatively low—but realistic—value of *η* ≈ 1%. Note, however, that higher values of *η* are feasible using objectives with higher NA and/or nanophotonic structures (e.g., solid immersion lenses). With the selected parameters according to Eq. (16) we estimate $\delta B\cong 20\, {{{\mathrm{nT}}}}/{\sqrt{{{\mathrm{Hz}}}}}$. Note that we deliberately consider the spectral linewidth to be constrained by the reciprocal *T*_2_* time and not by longer spin-echo *T*_2_ or dynamically decoupled spin-coherence times.

In this work, we have analyzed the spin properties of ${{{{\rm{V}}}}}_{{{{\rm{B}}}}}^{-}$ lattice defects in van der Waals hBN crystals in terms of their sensitivity to external perturbations and evaluated their advantages and disadvantages for possible applications as a nanoscale quantum sensor. The advantages include the simple intrinsic nature of the defect basically consisting of a missing boron atom, but also the potentially accessible very small distance between the sensor and the object to be sensed. In particular, we focused on the influence of temperature on the ground-state ZFS, which can be directly measured by cw ODMR and is explained by the temperature-dependent lattice compression/expansion. We showed that externally applied pressure induces lattice deformations and therefore can be mapped onto the defect ODMR spectrum of the ${{{{\rm{V}}}}}_{{{{\rm{B}}}}}^{-}$. However, temperature and pressure measurements need to be performed isobar or isothermal, respectively. Nevertheless, ${{{{\rm{V}}}}}_{{{{\rm{B}}}}}^{-}$ can be used for simultaneous magnetic field measurements with high sensitivity, due to the invariability of its g-factor with respect to temperature and pressure. By comparing three spin defect hosting solid systems, diamond, SiC, and hBN, we showed that the ${{{{\rm{V}}}}}_{{{{\rm{B}}}}}^{-}$ defect has comparable and, in some cases, even superior properties compared to 3D hosts. The coupling coefficient between ZFS and temperature in the temperature range 50–350 K is eight times larger than the corresponding factor for NV^−^ centers in diamond. Moreover, hBN is particularly interesting for temperature sensing under cryogenic conditions, i.e., at temperatures down to a few K, since ${{{{\rm{V}}}}}_{{{{\rm{B}}}}}^{-}$ centers exhibit a measurable temperature dependence of ZFS there, while it is nearly constant for NV^−^ centers in diamond. For completeness, a temperature sensor based on NV centers in diamond is still more sensitive, mainly due to stronger PL emission, higher ODMR contrast, and an optimized pulsed measurement protocol that exceeds the sensitivity of cw ODMR measurements. Regarding temperature sensing, we show that the heating effects due to laser excitation and absorbed microwave power are important, but they can be controlled. The resolution of ${{{{\rm{V}}}}}_{{{{\rm{B}}}}}^{-}$ to external magnetic fields is comparable to that of silicon vacancies in SiC, but lower than that of NV centers in diamond. However, we believe that the favorable optical properties of this 2D system, the recent demonstration of coherent control of ${{{{\rm{V}}}}}_{{{{\rm{B}}}}}^{-}$ spins in hBN together with the overcoming of inhomogeneous ODMR line broadening by multifrequency spectroscopy^20^ will stimulate the development of advanced pulse protocols^35^ and lead to a further increase in the resolution of this sensor. In addition, during the preparation of our manuscript, a relevant work on ${{{{\rm{V}}}}}_{{{{\rm{B}}}}}^{-}$ in hBN was submitted^36^ reporting an ODMR contrast of almost 10% and its high-temperature stability up to 600 K. Finally, the unique feature of hBN is its non-disturbing chemical and crystallographic compatibility with many different 2D materials, which gains a new fundamental functionality with the embedded spin centers and allows sensing in heterostructures with high resolution serving as a boundary itself.

## Methods

### Odmr

The low-field ODMR measurements are performed with a lab-built confocal microscope setup. A 532-nm laser (Cobolt Samba 100) is coupled into a 50-µm fiber and focused on the sample with a 10× objective (Olympus LMPLN10XIR), which excites an area on the sample with a diameter of about 10 μm. The photoluminescence is separated from the laser by a dichroic mirror and the remaining laser light is rejected by a 532-nm long-pass filter. The photoluminescence is then coupled into a 600 µm fiber and directed onto an avalanche photodiode (Thorlabs APD440A). A 0.5-mm wide copper strip-line is used to apply microwaves to the hBN sample placed on top. Microwaves from a signal generator (Stanford Research Systems SG384) are amplified by a Mini Circuits ZVE-3W-83+ amplifier. Lock-in detection is used (Signal Recovery 7230) by on−off modulation of the microwaves. For an external magnetic field, a permanent magnet is placed below the sample.

## Supplementary information

Supplementary Information

## Data Availability

All data needed to evaluate the conclusions in the paper are present in the main paper and the Supplementary Information.

## References

[1] Gruber A (1997). Scanning confocal optical microscopy and magnetic resonance on single defect centers. Science.

[2] Koehl WF, Buckley BB, Heremans FJ, Calusine G, Awschalom DD (2011). Room temperature coherent control of defect spin qubits in silicon carbide. Nature.

[3] Riedel D (2012). Resonant addressing and manipulation of silicon vacancy qubits in silicon carbide. Phys. Rev. Lett..

[4] Doherty MW (2014). Electronic properties and metrology applications of the diamond NV-center under pressure. Phys. Rev. Lett..

[5] Kraus H (2014). Magnetic field and temperature sensing with atomic-scale spin defects in silicon carbide. Sci. Rep..

[6] Dolde F (2011). Electric-field sensing using single diamond spins. Nat. Phys..

[7] Grinolds MS (2014). Subnanometre resolution in three-dimensional magnetic resonance imaging of individual dark spins. Nat. Nanotechnol..

[8] Kucsko G (2013). Nanometre-scale thermometry in a living cell. Nature.

[9] Schirhagl R, Chang K, Loretz M, Degen CL (2014). Nitrogen-vacancy centers in diamond: nanoscale sensors for physics and biology. Annu. Rev. Phys. Chem..

[10] Zhang W (2017). Depth-dependent decoherence caused by surface and external spins for NV centers in diamond. Phys. Rev. B.

[11] Shotan Z, Jayakumar H, Considine CR, Mackoit M, Fedder H (2016). Photoinduced modification of single-photon emitters in hexagonal boron nitride. ACS Photonics.

[12] Jungwirth NR (2016). Temperature dependence of wavelength selectable zero-phonon emission from single defects in hexagonal boron nitride. Nano Lett..

[13] Caldwell JD (2019). Photonics with hexagonal boron nitride. Nat. Rev. Mater..

[14] Hoese M (2020). Mechanical decoupling of quantum emitters in hexagonal boron nitride from low-energy phonon modes. Sci. Adv..

[15] Abdi M, Chou J-P, Gali A, Plenio MB (2018). Color centers in hexagonal boron nitride monolayers: a group theory and ab initio analysis. ACS Photonics.

[16] Gottscholl A (2020). Initialization and read-out of intrinsic spin defects in a van der Waals crystal at room temperature. Nat. Mater..

[17] Ivády V (2020). Ab initio theory negatively charged boron vacancy qubit hexagonal boron nitride.. npj Comp. Mater..

[18] Mendelson N (2021). Identifying carbon as the source of visible single-photon emission from hexagonal boron nitride. Nat. Mater..

[19] Chejanovsky, N. et al. Single spin resonance in a van der Waals embedded paramagnetic defect. *Nat. Mater*. 10.1038/s41563-021-00979-4 (2021). 10.1038/s41563-021-00979-4 33958771

[20] Gottscholl A (2021). Room temperature coherent control of spin defects in hexagonal boron nitride. Sci. Adv..

[21] Kianinia M, White S, Froch JE, Bradac C, Aharonovich I (2020). Generation of spin defects in hexagonal boron nitride. ACS Photonics.

[22] Gao X (2021). Femtosecond laser writing of spin defects in hexagonal boron nitride. ACS Photonics.

[23] Bradac C, Lim SF, Chang HC, Aharonovich I (2020). Optical nanoscale thermometry: from fundamental mechanisms to emerging practical applications. Adv. Opt. Mater..

[24] Paszkowicz W, Pelka JB, Knapp M, Szyszko T, Podsiadlo S (2002). Lattice parameters and anisotropic thermal expansion of hexagonal boron nitride in the 10–297.5 K temperature range. Appl. Phys. A.

[25] Chen XD (2011). Temperature dependent energy level shifts of nitrogen-vacancy centers in diamond. APL.

[26] Ivády V, Simon T, Maze JR, Abrikosov IA, Gali A (2014). Pressure and temperature dependence of the zero-field splitting in the ground state of NV centers in diamond: a first-principles study. Phys. Rev. B.

[27] Bosak A (2006). Elasticity of hexagonal boron nitride: inelastic X-ray scattering measurements. Phys. Rev. B.

[28] Lynch RW, Drickamer HG (1966). Effect of high pressure on the lattice parameters of diamond, graphite, and hexagonal boron nitride. J. Chem. Phys..

[29] Ivády V (2020). Ab initio theory of the negatively charged boron vacancy qubit in hexagonal boron nitride. npj Comput. Mater..

[30] Acosta VM (2010). Temperature dependence of the nitrogen-vacancy magnetic resonance in diamond. Phys. Rev. Lett..

[31] Doherty MW (2014). Temperature shifts of the resonances of the NV^−^ center in diamond. Phys. Rev. B.

[32] Hayashi K (2018). Optimization of temperature sensitivity using the optically detected magnetic-resonance spectrum of a nitrogen-vacancy center ensemble. Phys. Rev. Appl..

[33] Zhang N (2018). Microwave field uniformity impact on DC magnetic sensing with NV ensembles in diamond. IEEE Sens. J..

[34] Acosta VM (2009). Diamonds with a high density of nitrogen-vacancy centers for magnetometry applications. Phys. Rev. B.

[35] Soltamov VA (2019). Excitation and coherent control of spin qudit modes in silicon carbide at room temperature. Nat. Commun..

[36] Liu, W., et al. Temperature-dependent energy-level shifts of spin defects in hexagonal boron nitride. *ACS Photonics* (2021). 10.1021/acsphotonics.1c00320.

