# Supplementary material for: Spin defects in hBN as promising temperature, pressure and magnetic field quantum sensors
Source: Nat Commun. 2021 Jul 22;12:4480. doi: 10.1038/s41467-021-24725-1 (PMC8298442; doi:10.1038/s41467-021-24725-1)
Supplement: Supplementary file 1 — Supplementary Information [file 41467_2021_24725_MOESM1_ESM.pdf]

Supplementary Information
for

Spin defects in hBN as promising temperature, pressure and magnetic field quantum sensors

Andreas Gottscholl[1], Matthias Diez[1], Victor Soltamov[1,2], Christian Kasper[1], Dominik Krauße[1], Andreas Sperlich[1], Mehran Kianinia[3, 4], Carlo Bradac[5], Igor Aharonovich[3, 4], Vladimir Dyakonov[1]*

[1] Experimental Physics 6 and Würzburg-Dresden Cluster of Excellence ct.qmat, Julius Maximilian University of Würzburg, 97074 Würzburg, Germany
[2] Ioffe Institute, St. Petersburg 194021, Russia
[3] School of Mathematics and Physical Sciences, University of Technology Sydney, Ultimo, NSW 2007, Australia
[4.]Centre of Excellence for Transformative Meta-Optical Systems, University of Technology Sydney, Ultimo, NSW 2007, Australia
[5] Department of Physics & Astronomy, Trent University, 1600 West Bank Dr., Peterborough 28 ON, K9J 0G2, Canada

*email: vladimir.dyakonov@uni-wuerzburg.de

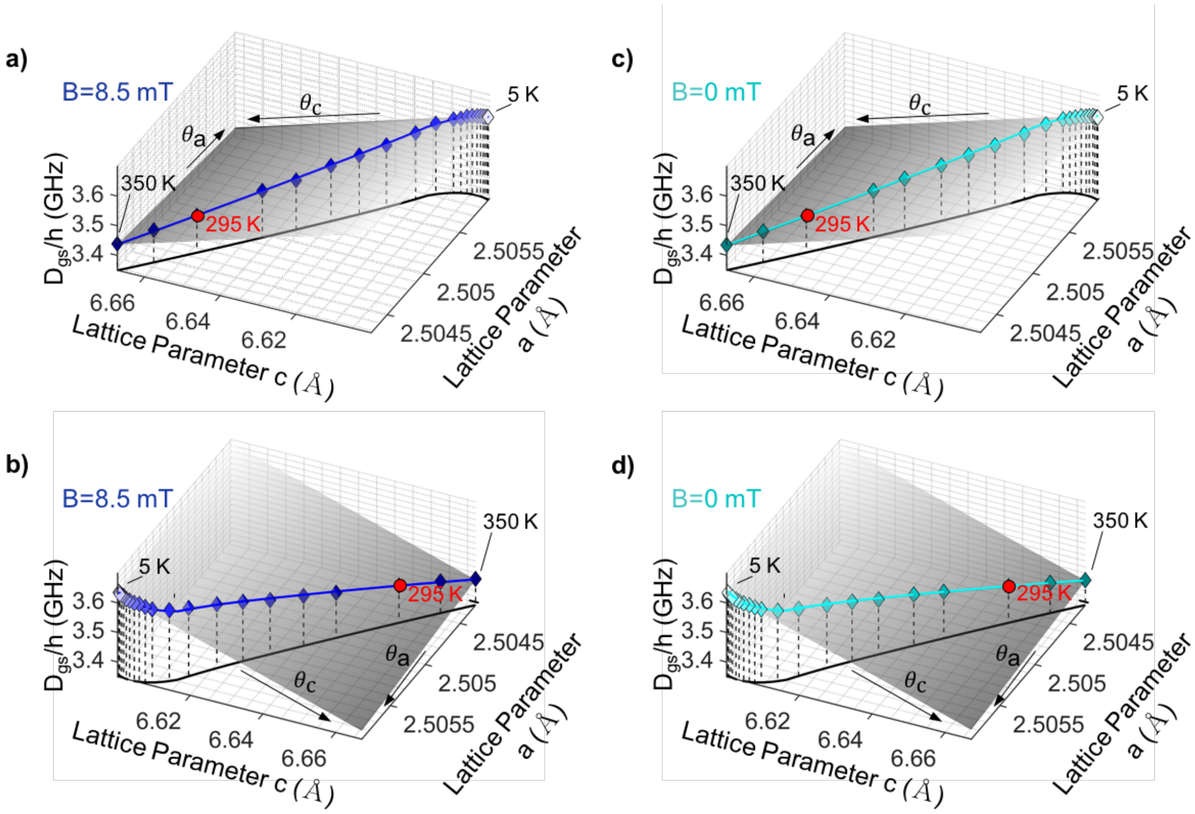

Supplementary Figure 1 Zero-field splitting dependence on the lattice parameters a **and c.** Eq. (2) is fitted (solid lines) to the experimental data displayed as diamonds. The ZFS reference temperature *T* = 295 K is marked by the red dot. The assignment of colors of fitting lines (blue for *B* = 8.5 mT and cyan for 0 mT) is the same as in Fig. 2. Fitting Eq. (2) to the 3-dimensional data set $D_{gs}(\mathrm{a}, c, T)$ allows estimating the slopes θ_{a} *and* θ_c. **a**, **b** *B* = 8.5 mT dataset with corresponding fit. **b** is rotated by 180° relative to **a** to illustrate the 3-dimensional shape of the fit function. **c**, **d** *B* = 0 mT dataset with corresponding fit with the same rotation of 180° between **c** and **d.**

Calculation of polynomial coefficients of Table 1 for temperature measurements

In the main text we derive a relationship between the ZFS $D_{gs}(\eta_{\mathrm{a}},\eta_c)$ and the relative changes η_{a},η_c of the temperature-dependent lattice parameters $\mathrm{a}(T)$ *and* $c(T)$ (see Eq. (2) in the main text). In the following, we will extend this and derive an expression to compute $D_{gs}(T)$ directly. First, the temperature-dependent lattice parameters $\mathrm{a}(T)$ *and* $c(T)$ are required. We use the mathematical description in terms of a third-order polynomial as described in Ref. [24].

$$\mathrm{a}(T) = \sum_k A_k^{\mathrm{a}} T^k \; (k = 0,1,2,3) \tag{1}$$
$$c(T) = \sum_k A_k^{c} T^k \; (k = 0,1,2,3) \tag{2}$$

A_k^{a} and A_k^{c} are the polynomial coefficients given in Table 3 of Ref. [24] and k is an integer. The relative changes of the lattice parameters with respect to room temperature are:

$$\eta_{\mathrm{a}}(T) = \frac{\mathrm{a}(T)-\mathrm{a}(295\,K)}{\mathrm{a}(295\,K)} = \frac{\mathrm{a}(T)}{\mathrm{a}(295\,K)} - 1 \tag{3}$$
$$\eta_c(T) = \frac{c(T)-c(295\,K)}{c(295\,K)} = \frac{c(T)}{c(295\,K)} - 1 \tag{4}$$

Inserting the polynomials of Supplementary Eq. (1) and Supplementary Eq. (2) into Supplementary Eq. (3) and Supplementary (4) results in

$$\eta_{\mathrm{a}}(T) = \frac{A_0^{\mathrm{a}}}{\mathrm{a}(295\,K)} - 1 + \frac{A_1^{\mathrm{a}}}{\mathrm{a}(295\,K)}T + \frac{A_2^{\mathrm{a}}}{\mathrm{a}(295\,K)}T^2 + \frac{A_3^{\mathrm{a}}}{\mathrm{a}(295\,K)}T^3 \tag{5}$$
$$\eta_c(T) = \frac{A_0^{c}}{c(295\,K)} - 1 + \frac{A_1^{c}}{c(295\,K)}T + \frac{A_2^{c}}{c(295\,K)}T^2 + \frac{A_3^{c}}{c(295\,K)}T^3. \tag{6}$$

These expressions are grouped in polynomials with coefficients $A_k^{\eta_{\mathrm{a},c}}$:

$$\eta_{\mathrm{a}}(T) = \sum_k A_k^{\eta_{\mathrm{a}}} T^k \quad \text{and} \quad \eta_c(T) = \sum_k A_k^{\eta_c} T^k \tag{7}$$
$$A_0^{\eta_{\mathrm{a}}} = \frac{A_0^{\mathrm{a}}}{\mathrm{a}(295\,K)} - 1 \quad \text{and} \quad A_0^{\eta_c} = \frac{A_0^{c}}{c(295\,K)} - 1 \quad \text{for k=0} \tag{8}$$
$$A_k^{\eta_{\mathrm{a}}} = \frac{A_k^{\mathrm{a}}}{\mathrm{a}(295\,K)} \quad \text{and} \quad A_k^{\eta_c} = \frac{A_k^{c}}{c(295\,K)} \quad \text{for k= 1,2,3} \tag{9}$$

Next, we will implement $\eta_{\mathrm{a}}(T)$ and $\eta_c(T)$ into Eq. (2) of the main text:

$$D_{gs}(\eta_{\mathrm{a}},\eta_c) = D_{gs,295K} + \theta_{\mathrm{a}}\eta_{\mathrm{a}}h + \theta_c\eta_c h \tag{10}$$

Finally, this results in a polynomial for the ZFS $D_{gs}(T)$ with coefficients A_k^D that are directly calculated from the polynomial coefficients A_k^{a} and A_k^{c} for the temperature-dependent lattice compression [24] together with reference parameters at T = 295 K: a(295K) = 2.5047 Å, c(295K) = 6.6532 Å, $D_{gs,295K}/h$ = 3.48 GHz.

$$D_{gs}(T) = h\sum_k A_k^D T^k \tag{11}$$
$$A_0^D = D_{gs,295K}/h + \theta_{\mathrm{a}}\left(\frac{A_0^{\mathrm{a}}}{\mathrm{a}(295\,K)} - 1\right) + \theta_c\left(\frac{A_0^{c}}{c(295\,K)} - 1\right) \quad \text{for k=0} \tag{12}$$
$$A_k^D = \theta_{\mathrm{a}}\frac{A_k^{\mathrm{a}}}{\mathrm{a}(295\,K)} + \theta_c\frac{A_k^{c}}{c(295\,K)} \quad \text{for k= 1,2,3} \tag{13}$$

Since A_k^{a} and A_k^{c} are sectionally defined for different temperature ranges, we obtain the same sections for the coefficients A_k^D. The results are shown in Supplementary Table 1 (equal to Table 1 in the main text).

Temperature range (K)	A_0^D (GHz)	A_1^D (MHz K^{-1})	A_2^D (kHz K^{-2})	A_3^D (Hz K^{-3})
5-128	3.6367	0	-4.4308	11.468
128-189	3.6109	0.22839	-3.8805	5.522
189-350	3.6664	-0.55659	-0.2383	0

Supplementary Table 1. *Calculated polynomial coefficients A_k^D for Supplementary Eq. (11). The three temperature ranges arise from the sectionally defined polynomial for the temperature-dependent lattice parameters* a *and* c [24].

To assess the heating effect induced by the laser, we measured ODMR spectra at different laser powers between 200 and 1000 mW. An overall shift of <10 MHz due to laser heating is observed. The data is shown in Supplementary Figure 2.

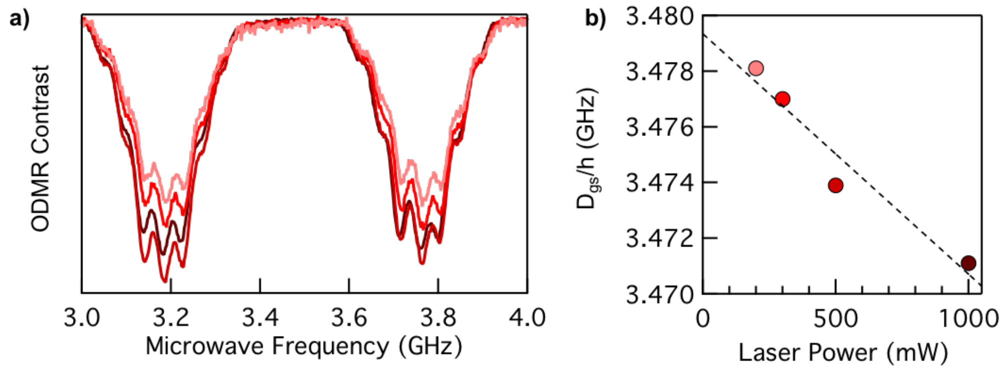

Supplementary Figure 2 Influence of laser power on the ODMR signal. a cw-ODMR signals for different laser powers. **b** Change of the ZFS parameter D_{gs}/h as a function of the applied laser power. A total shift of <10 MHz due to laser heating is observed. This effect can be neglected for other measurements, since all other measurements presented in this work were performed with laser power <100 mW.

To estimate the heating induced by the microwaves, we measured ODMR spectra at different microwave power between 0.03 and 3 W at room temperature (ambient laboratory conditions). We summarize this data in Supplementary Figure 3. We do observe a change of the ZFS of about 5 MHz, which corresponds to a heating of 10K (red symbols). However, we tend to assign the main effect to the resistive heating of the stripline and not to the power absorbed by the spin system in resonance. The two effects can be separated by a precise measurement in a stabilized cryo-system (shown in purple). The local sample temperature is constant within the error bars, which means that the resonant absorbed microwaves have very little or no influence on the temperature dependent ZFS in this microwave power range.

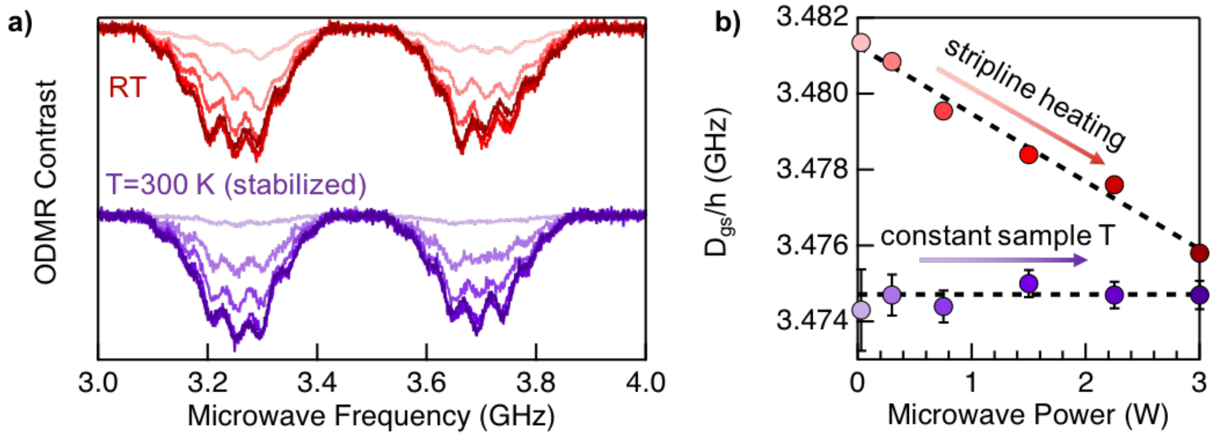

Supplementary Figure 3 Influence of microwave power on the ODMR signal at room temperature (red) and for temperature stabilized stripline at 300 K (purple). a cw-ODMR signals for different microwave powers. The spectra without temperature stabilization (red) are shifted towards lower frequencies for higher microwave powers. **b** Change of the ZFS parameter D_{gs}/h as a function of the applied microwave power. A total shift of 5 MHz due to the resistive heating of the stripline is observed (red spheres). However, the local sample temperature (purple spheres) remains unaffected. The error bars of the D_{gs} value represent the standard deviation of fits to spectrum.

For pressure sensing, we applied sinusoidal modulation to the B-field about a fixed value. To optimize sensitivity, we measured the influence of modulating the amplitude of the magnetic field onto the ODMR signal to select the one with best S/N ratio. This is shown in Supplementary Figure 4. It is interesting to note, that this modulation scheme also allows us to resolve the hyperfine structure resulting from having 3 equivalent nitrogen nuclei surrounding the boron vacancy.

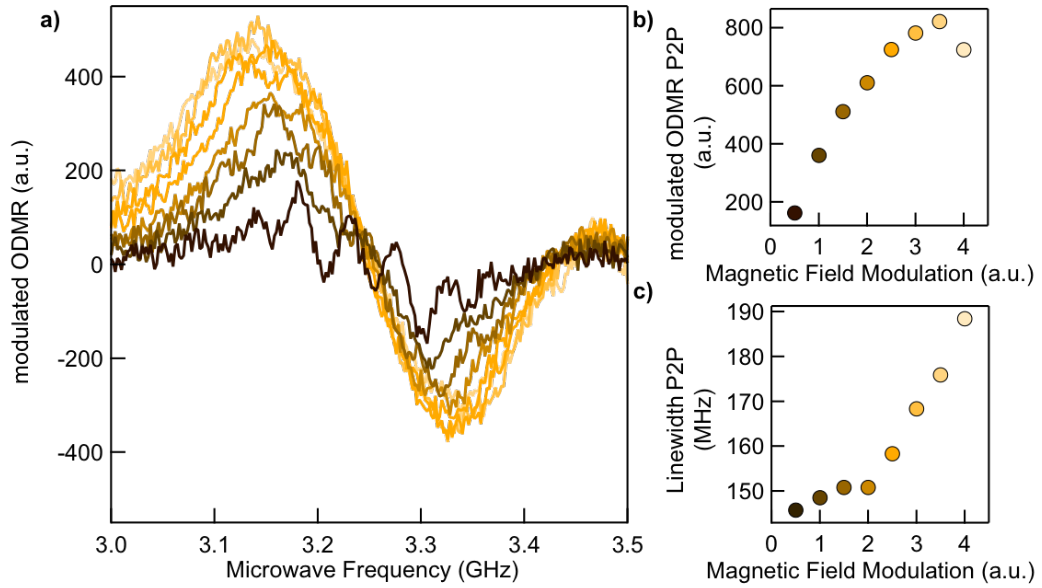

Supplementary Figure 4 Influence of the magnetic field modulation on the ODMR spectra. a Magnetic field modulation leads to the first derivative of the standard cw-ODMR signal. **b** Peak-to-peak amplitude can be enhanced by increasing the magnetic field modulation. **c** ODMR linewidth vs. modulation amplitude. The overmodulation results in line broadening of the signal.

To address possible influence of local strains, we performed ODMR experiments at different sites on the sample and at different temperatures. The parameter we monitored was the off-axis ZFS E_{gs}, sometimes called strain parameter, which we show in Supplementary Figure 5. As can be seen, E_{gs}/h does not depend on the local spot on the sample, but scatters around E_{gs}/h=49 MHz at different temperatures.

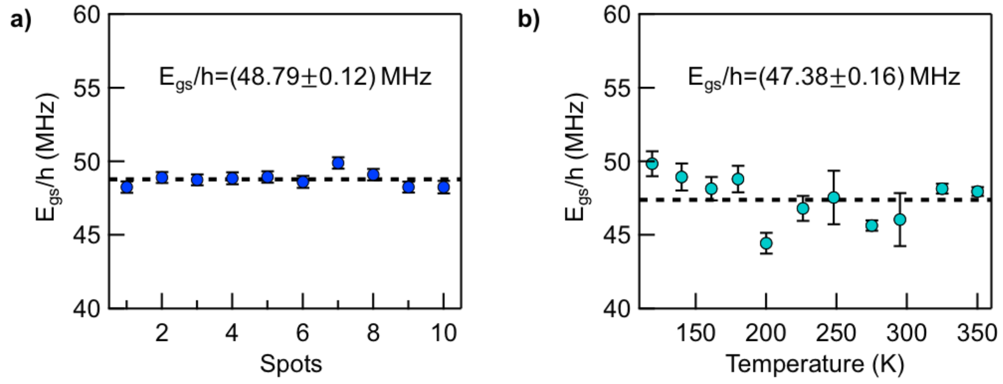

Supplementary Figure 5 Off-axis zero-field splitting term E_{gs}/h measured at different sites on the sample **a** and at different temperatures (110-350K) **b** for a selected site. The E_{gs}/h values are scattered around 49 MHz, but without a clear trend. The error bars are the same as defined in Supplementary Fig. 3b.
